# Shade‐induced reduction of stem nonstructural carbohydrates increases xylem vulnerability to embolism and impedes hydraulic recovery in *Populus nigra*

**DOI:** 10.1111/nph.17384

**Published:** 2021-05-15

**Authors:** Martina Tomasella, Valentino Casolo, Sara Natale, Francesco Petruzzellis, Werner Kofler, Barbara Beikircher, Stefan Mayr, Andrea Nardini

**Affiliations:** ^1^ Dipartimento di Scienze della Vita Università di Trieste Via L. Giorgieri 10 Trieste 34127 Italy; ^2^ Dipartimento di Scienze Agroalimentari Ambientali e Animali Università di Udine Via delle Scienze 91 Udine 33100 Italy; ^3^ Department of Botany University of Innsbruck Sternwartestraße 15 Innsbruck 6020 Austria

**Keywords:** black poplar, carbon starvation, hydraulic recovery, nonstructural carbohydrate (NSC), shading, xylem anatomy, xylem sap pH, xylem vulnerability

## Abstract

- Nonstructural carbohydrates (NSCs) have been suggested to affect xylem transport under fluctuating water availability, but conclusive evidence is still lacking. We tested the effect of shade‐induced NSC depletion on xylem vulnerability to embolism and hydraulic recovery on *Populus nigra* saplings.
- Vulnerability was assessed in light‐exposed (L) and shaded (S) plants with the hydraulic method, and *in vivo* with the optical method and X‐ray micro‐computed tomography. Plants were stressed to 80% loss of hydraulic conductance (PLC) and re‐irrigated to check for possible recovery. We measured PLC, bark and wood NSC content, as well as xylem sap pH, surface tension (*γ*_sap_) and sugar concentration, before, during and after drought.
- Shading induced depletion of stem NSC (mainly starch) reserves. All methods converged in indicating higher xylem vulnerability in S than in L plants. This difference was not explained by xylem vessel and pit anatomy or by *γ*_sap_. Shading impeded sap acidification and sugar accumulation during drought in S plants and prevented hydraulic recovery, which was observed in L plants.
- Our results highlight the importance of stem NSCs to sustain xylem hydraulic functioning during drought and suggest that light and/or adequate stem NSC thresholds are required to trigger xylem sap chemical changes involved in embolism recovery.

## Introduction

Drought‐induced xylem embolism leads to loss of plant hydraulic conductance, eventually up to critical levels causing hydraulic failure, hindering hydration of cells and tissues and leading to plant death (Tyree & Sperry, 1988). Xylem vulnerability to embolism depends on biophysical factors related to wood anatomy (conduit and pit characteristics (Lens *et al*., 2011; Gleason *et al*., 2016; Li *et al*., 2016; Hacke *et al*., 2017); conduit connectivity (Mrad *et al*., 2018)) and to xylem sap surface tension (*γ*_sap_), which affects the air‐seeding threshold and is determined by sap physical‐chemical properties (Tyree & Zimmermann, 2002). Xylem sap properties can change on daily and seasonal scales, eventually influencing xylem hydraulics (e.g. Nardini *et al*., 2011a; Losso *et al*., 2017).

Hydraulic failure is a major cause of drought‐induced tree mortality (Nardini *et al*., 2013; Adams *et al*., 2017), and seems to be favoured by depletion of nonstructural carbohydrates (NSCs) (McDowell *et al*., 2008, 2011). Indeed, several studies have suggested the importance of NSCs in sustaining plant hydraulic function and survival under drought (Sala *et al*., 2012; O’Brien *et al*., 2014; Sevanto *et al*., 2014; Tomasella *et al*., 2019c). However, whether and how NSCs may influence xylem hydraulics under water availability fluctuations *per se* remains an open question.

Stems and roots are not only essential organs for water uptake and supply to the leaves, but also the main NSC storage sites in woody plants (Hartmann & Trumbore, 2016). Fluctuations of NSC content in woody tissues and in xylem sap can occur daily (Tixier *et al*., 2018), seasonally (Richardson *et al*., 2013), and at the onset of several stressors such as drought, freeze–thaw events and pathogen infection (Secchi & Zwieniecki, 2012; Ito *et al*., 2013; Hartmann & Trumbore, 2016; Tomasella *et al*., 2017; Paljakka *et al*., 2020). During physiological drought, in particular, sugars contribute with inorganic ions and amino acids to the maintenance of cell turgor through osmoregulation (Morgan, 1984; Chaves *et al*., 2003; Blum, 2017). Sugars in the stem derive from stem photosynthesis, local starch hydrolysis or long‐distance phloem transport. Xylem and phloem are in contact via radial parenchyma cells, so that water and solutes can be exchanged between these two compartments (Zweifel *et al*., 2000; Hölttä *et al*., 2009; Sevanto *et al*., 2014; Pfautsch *et al*., 2015).

Recent studies have highlighted the possible roles of stem NSCs in sustaining xylem hydraulics. Shading branches in several mangrove species reduced stem hydraulic conductivity (Schmitz *et al*., 2012), invoking a possible connection with local starch depletion. Similarly, shaded stems of *Populus nigra* saplings showed earlier onset of acoustic emissions during drought (De Baerdemaeker *et al*., 2017) and another study on *Populus tremula* underlined the importance of woody tissue photosynthesis in sustaining NSC pools and maintaining stem hydraulic integrity during drought (De Roo *et al*., 2020). However, the link between hydraulic safety and NSC availability still awaits further experimental evidence and mechanistic explanation (Tomasella *et al*., 2019c).

Several studies have drawn attention to the importance of NSCs for post‐drought hydraulic recovery (Savi *et al*., 2016; Trifilò *et al*., 2017; Tomasella *et al*., 2019a). Nonstructural carbohydrates are required for building new functional xylem by cambial activity over the mid‐to‐long term (Cochard *et al*., 2001; Brodribb *et al*., 2010), and for relatively fast (hours to days) osmotically driven refilling of embolized conduits (e.g. Salleo *et al*., 2004; Nardini *et al*., 2011; Brunetti *et al*., 2020; Secchi *et al*., 2021). Albeit controversial in several species, the biology and chemistry of embolism repair have been accurately described in poplars, and a mechanistic model has been proposed to explain the process (Secchi & Zwieniecki, 2012; Pagliarani *et al*., 2019). Starch depletion in wood parenchyma cells during drought has been reported in several species (e.g. Salleo *et al*., 2009; Secchi & Zwieniecki, 2011; Tomasella *et al*., 2019b), together with a decrease in xylem sap pH that would prime the accumulation of sugars in the xylem apoplast, consequently creating an osmotic gradient to refill xylem conduits upon drought relief (Secchi & Zwieniecki, 2016). Accordingly, the experimental reduction of metabolic activities responsible for sugar accumulation in xylem sap under drought led to delayed hydraulic recovery after re‐irrigation (Secchi *et al*., 2021). However, proof that adequate stem NSC pools are essential for modulation of xylem sap composition during drought is missing, and the impact of stem NSC concentration on hydraulic recovery requires further testing (Tomasella *et al*., 2019c). Indeed, refilling of embolized conduits in stem segments of *Salix* *matsudana* submerged in water was possible only in combination with NSC accumulation as a result of light exposure (Liu *et al*., 2019), and high stem NSC pools were the prerequisite for the occurrence of hydraulic recovery in *Fraxinus ornus* saplings (Tomasella *et al*., 2019a).

We conducted a drought‐recovery experiment on *P*. *nigra* saplings that underwent prolonged (50 d) shading, aimed at depleting NSC reserves. We aimed to test the effects of NSC depletion on xylem vulnerability to embolism and hydraulic recovery. We first compared three different methods to quantify xylem embolism, that is, a destructive hydraulic method and two *in vivo* imaging methods (optical and X‐ray micro‐computed tomography (micro‐CT)). We aimed to cross‐validate methods, and to compare the hydraulic vulnerabilities between shaded and light‐exposed plants. We also quantified several stem features that might be relevant for short‐term changes in xylem vulnerability to embolism: xylem anatomy (conduit and intervessel pit characteristics), wood and bark NSC concentrations, and xylem sap properties (*γ*_sap_, NSC concentration, pH). Finally, we investigated the effects of NSC depletion on short‐term hydraulic recovery after drought relief. Following the proposed mechanistic model of xylem hydraulic recovery, a low NSC (particularly starch) content in the stem could impede high sucrose accumulation in the symplast during drought and its subsequent efflux into the xylem apoplast through sucrose‐proton symporters. The sugars obtained from the leftover starch in NSC‐depleted plants could also be preferentially allocated to living cells for other essential functions such as osmoregulation for turgor maintenance under well‐hydrated and, most importantly, under drought conditions. The lack of proton efflux to the xylem apoplast would also impede its acidification and the subsequent cascade of events inducing the accumulation of monosaccharides in the apoplast (Secchi & Zwieniecki, 2012). Consequently, the sugar concentration in the xylem sap would be too low to reclaim water for the refilling process upon stress relief. We specifically tested the hypotheses that shading increases xylem vulnerability in parallel with NSC depletion, which, in turn, would impede a decrease in pH and sugar accumulation in the xylem sap during drought, and reduce/inhibit short‐term hydraulic recovery after drought.

## Materials and Methods

### Plant material and experimental design

On 20 March 2019, 120 *Populus nigra* L. saplings (3 yr old) obtained from rooted cuttings were provided by a public nursery in 1 l pots (Vivai Pascul, Regional Forestry Service, Tarcento, Italy) and transplanted in 3.5 l pots filled with a substrate commonly used in green roof installations (total porosity = 67%, plant available water content = 0.34 g g^−1^; Savi *et al*., 2013). Basal shoot diameter and tree height at transplant (before spring flush) were 6.3 ± 0.1 mm and 92.0 ± 1.5 cm, respectively. We are aware of possible effects of the relatively small pot size on plant growth and underlying physiological responses (Poorter *et al*., 2012). However, the pots used provided sufficient volume for root growth (see the Results section) and this factor was unlikely to interfere with the mechanistic aims of the experiment. Water was supplied twice a day via drip irrigation, and pots were irrigated to field capacity until drought was applied. Pots were randomly and periodically shifted within the greenhouse to assure plants exposure to uniform growth conditions. A slow release and a liquid fertilizer (Cifo Srl, S. Giorgio di Piano, Italy), insecticides and a fungicide were supplied in the spring, before applying the shading treatment (see later).

**Fig. 1:**
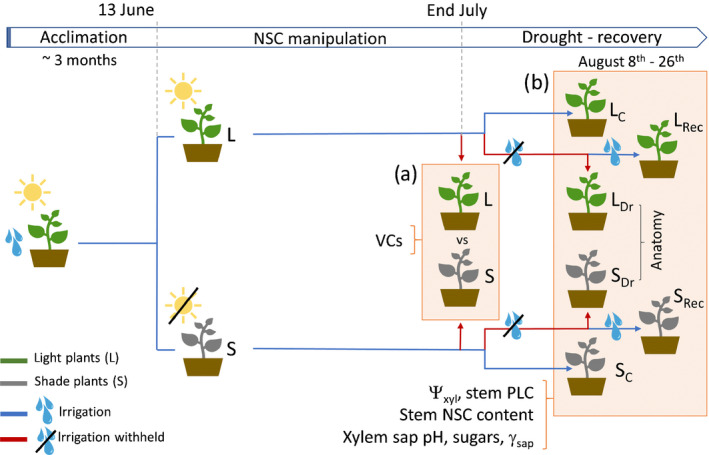
Experimental design. Light‐exposed (L) and shaded (S) *Populus nigra* plants are shown in green and grey, respectively. Blue and red lines indicate irrigation and drought (no irrigation) periods, respectively. Orange boxes highlight the two main parts of the experiment: hydraulic vulnerability assessment through hydraulic and optical vulnerability curves (VCs; a); drought‐recovery experiment comparing control (L_C_, S_C_), drought (L_Dr_, S_Dr_) and recovery (L_Rec_, S_Rec_) plants (b). Arrows indicate harvests. The main measurements performed are shown next to the curly brackets.

**Fig. 2:**
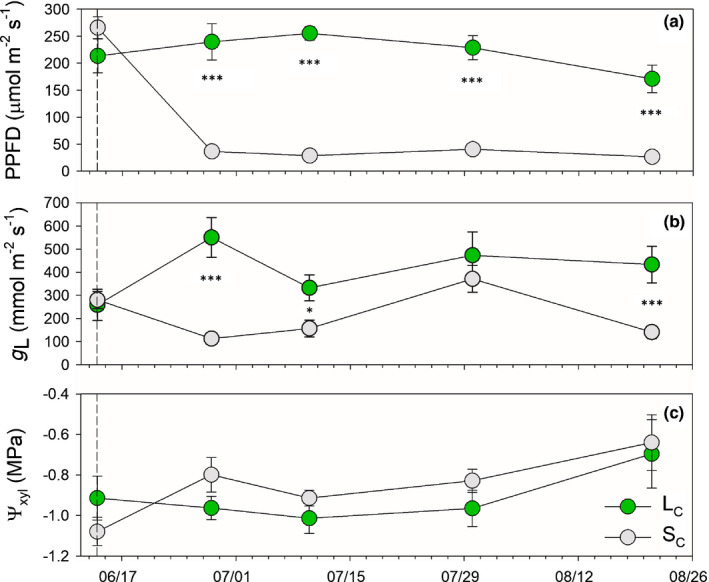
Photosynthetic photon flux density (PPFD, a), leaf conductance to water vapour (*g*_L_, b) and xylem water potential (Ψ_xyl_, c) measured in control light (L_C_) and shade (S_C_) *Populus nigra* plants during the shading period at midday. Values are means ± SE. The dashed vertical line indicates the beginning of the shading treatment (the first measuring campaign was performed before shading). *, 0.01 < *P* < 0.05; ***, *P* < 0.001. *x*‐axis date style: month/day.

Fig. 1 illustrates the experimental design. On 13 June (after 3 months of acclimation to glasshouse conditions) plants were randomly divided in two groups. One group was maintained under glasshouse light (L, light treatment), whereas the other was placed under a shading net reducing photosynthetic photon flux density (PPFD, μmol m^−2^ s^−1^) by 80–90% (S, shade treatment), while ensuring proper aeration. Average PPFD values in the glasshouse at midday were 221 ± 16 and 33 ± 3 μmol m^−2^ s^−1^ in the L and S treatments, respectively (Fig. 2a). In S plants average midday PPFD was slightly above the light compensation point for poplars (Zhao *et al*., 2015).

**Fig. 3:**
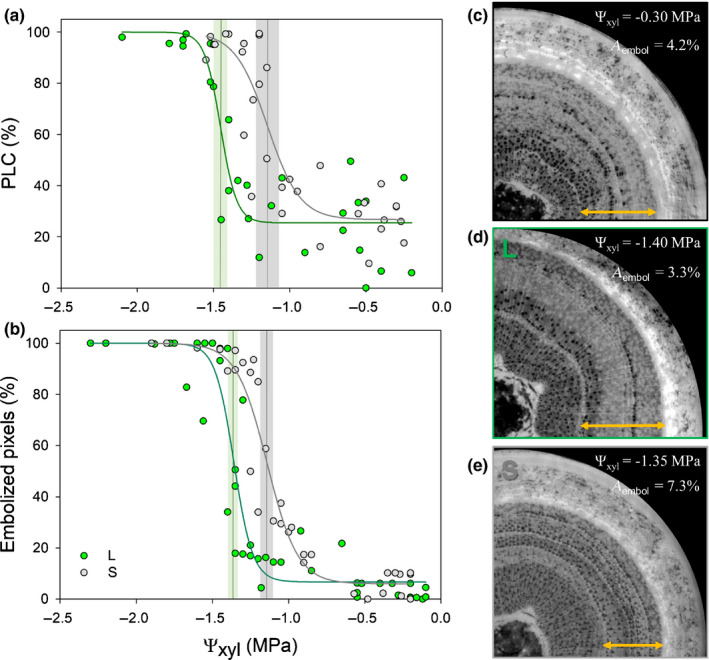
Stem xylem vulnerability curves assessed with the hydraulic method in cut stems from light (L, green) and shaded (S, grey) *Populus nigra* plants dried in pots (a) and with the optical method in intact plants (b); representative transverse images of 3‐ to 4‐yr‐old stems obtained with micro‐CT in intact well‐hydrated plants (c) and in L (d) and S plants (e) stressed to *c*. −1.4 MPa. PLC, percentage loss of hydraulic conductance. Vertical lines and shaded areas show the inflection point of the curve (*x*0) and the 95% confidence interval, respectively. In (c–e) Ψ_xyl_ and the percentage of embolized sapwood area (*A*_embol_), calculated excluding the older year ring (yellow arrows indicate the considered xylem portion), are reported and air‐filled conduits are shown in black.

USB dataloggers (EL‐USB‐2, Lascar Electronics Inc., Salisbury, UK) were placed at canopy height to monitor air temperature and relative humidity (RH) on an hourly basis. From the beginning of shading treatment to final harvest, mean daily temperature was 26.9 ± 0.2°C and 27.2 ± 0.2°C, and RH was 59 ± 1% and 63 ± 1% for L and S groups, respectively (Supporting Information Fig. S1). At the end of July (*c*. 50 d of shading), 40 plants per group were subjected to drought by withholding irrigation. From late July to early August, 26–28 stressed plants per group were harvested at different xylem water potential (Ψ_xyl_, MPa), to cover the range necessary for constructing hydraulic vulnerability curves (see later). The remaining 12–14 plants per group were then stressed to the respective target Ψ_xyl_ at 80% loss of hydraulic conductance (PLC; i.e. at *c*. −1.5 and −1.25 MPa in L and S plants, respectively; see Fig. 3 and later), which was reached in 6–10 d in L plants and 9–15 d in S plants, as a result of lower water consumption in the latter group. Then, six to seven drought‐stressed plants (L_Dr_ and S_Dr_ hereafter) were harvested for hydraulic and NSC measurements. The remaining six to seven plants (recovery) were re‐irrigated to field capacity (L_Rec_ and S_Rec_) and harvested 3 d later to assess possible hydraulic recovery. A group of well‐watered (control) plants (L_C_ and S_C_, *n* = 10 in total) was harvested and measured within the same period as the drought and recovery plants. Harvest of L_Dr_, S_Dr_, L_Rec_ and S_Rec_ plants started on 8 August and ended on 26 August. Plants were always harvested between 12:00 and 14:30 h to avoid NSC diurnal fluctuation effects (Tixier *et al*., 2018).

### Water relations

Midday Ψ_xyl_ and leaf conductance to water vapour (*g*_L_, mmol s^−1^ m^−2^) were measured in randomly selected plants (*n* = 7 per date and treatment) before applying the shading treatment (14 June), and every 2 wk during the shading period. Because the glasshouse was not equipped with artificial light, measurements were performed on sunny days between 12:00 and 14:00 h. Ψ_xyl_ was measured with a pressure chamber (mod. 1505D, PMS Instrument co., Albany, OR, USA) on one mature leaf per plant, previously wrapped in cling film and aluminium foil (at 9:00 h) to stop transpiration and favour equilibration between leaf and stem xylem. *g*_L_ was measured with a steady‐state porometer (SC‐1; Decagon Devices Inc., Pullman, WA, USA) on one light‐exposed leaf from the same plant used for Ψ_xyl_ measurements. Additional Ψ_xyl_ were measured on each plant immediately before hydraulic measurements, and for optical vulnerability curves.

### Stem PLC quantification and hydraulic vulnerability curves

Percentage loss of hydraulic conductance was measured in stem segments of the 1 yr growth stem portion in L and S plants at Ψ_xyl_ between 0 and −2.0 MPa, to generate hydraulic vulnerability curves (VCs). PLC was also measured in L_Dr_, S_Dr_ and re‐irrigated plants (L_Rec_ and S_Rec_) to check for possible hydraulic recovery. After measuring Ψ_xyl_, whole pots wrapped in a plastic bag were immersed in a bucket filled with tap water, and the stem was cut at the base under water. The stem was transferred in clean tap water and a second cut was made at *c*. 30 cm from the initial cut (i.e. twice the maximum vessel length, as checked during preliminary analysis). The basal stem segment was quickly dried with paper towel and used for xylem sap extraction (see below). The distal segment was trimmed at both ends under clean tap water with 1‐cm‐long consecutive cuts until a segment 6–7 cm long was obtained. After clear thin cuts made with a sharp razor blade at both ends, the segment was used for hydraulic conductivity measurements. A 4 cm segment next to it was used for NSC analysis.

Hydraulic conductance was measured with an hydraulic apparatus (Tomasella *et al*., 2019a), using filtered (0.45 µm) and degassed mineral water added with 10 mM KCl as perfusion solution (Nardini *et al*., 2007). Stem hydraulic conductance was measured gravimetrically under a water head of 4 kPa before (initial hydraulic conductance, *k*_i_) and after (maximum hydraulic conductance, *k*_max_) flushing the sample at high pressure (0.15 MPa) for 5 min to remove embolism.

PLC was calculated as:

$$\text{PLC}=100\times [1‐(k_{i}/k_{\max})]$$

### *In vivo* optical vulnerability curves

When drought was applied to the other plants (i.e. after *c*. 50 d of shading treatment), three to four well‐watered plants per group (L and S) were sampled to construct optical stem VCs (Brodribb *et al*., 2017). A Raspberry Pi computer (http://www.raspberrypi.org) was connected to an 8 megapixel camera enclosed in a 3D‐printed clamp. Ten to 14 leaves per plant were wrapped in plastic bags covered with aluminium foil for Ψ_xyl_ measurements. Intact plants were placed on a bench and a small area of bark (*c*. 25 × 5 mm) of current‐year shoot was gently removed without damaging the xylem, which was covered with a thin layer of ultrasound transmission gel (Ecoultragel; Pirrone Srl, Milan, Italy) to reduce evaporation. The stem was fixed at the camera clamp, and image capturing started at the same time as the first Ψ_xyl_ measurement (Ψ_xyl_ > − 0.3 MPa). Plants were left to dry under laboratory conditions. Pictures were recorded every 5 min, and Ψ_xyl_ was measured at regular intervals. Image capture continued until no embolism events were detected for at least 6 h. Images were processed with imagej (US National Institutes of Health, http://imagej.nih.gov/ij/) to quantify embolized pixel area. The embolized pixel area was calculated as a fraction of total exposed xylem area. The percentage of embolized pixels was then calculated with respect to the maximum embolized pixel area fraction, and related to Ψ_xyl_ measured at the time of image capture (https://github.com/OpenSourceOV).

### X‐ray micro‐CT embolism quantification

Hydraulic and optical VCs were validated via micro‐CT imaging of stems in intact plants. Measurements were performed at the SYRMEP beamline, Elettra Sincrotrone Trieste (www.elettra.trieste.it) on 13–15 September 2019. Owing to the *c*. 1 month delay with respect to optical and hydraulic measurements, six L_C_ plants were put under the shading net on 29 July, and shading was maintained for the same time interval as in the main experiment. Two plants in each of the following treatments were scanned: well‐watered plants (L_C_ and S_C_), and plants drought‐stressed to a Ψ_xyl_ of *c*. −1.4 MPa at which, according to the hydraulic and optical methods, PLC was different between the two groups (see Fig. 3a,b). The CT setup at the beamline allowed only the basal stem (3–4 yr old) to be scanned. After measuring Ψ_xyl_, stems were quickly fixed to the sample holder and the plant was wrapped in clingfilm to avoid water loss. The average X‐ray source energy was 22 keV and pixel size was 3 μm. Tomographic reconstructions of the 2048 images, acquired during 180° rotation of the sample, were performed with the software syrmep tomoproject (Brun *et al*., 2015). Images were processed with imagej, setting pixel value thresholds to highlight the embolized vessels and using the particle analysis function. Owing to the larger diameter of stems compared with the field of view (6 × 6 mm), analyses were conducted in half of the stem section. The percentage of embolized sapwood area (*A*_embol_) was calculated, as this has been shown to correlate strongly to PLC calculated from the Hagen–Poiseuille equation in micro‐CT images of poplar stems (Secchi *et al*., 2021). The inner (older) year‐ring was not included in calculations, as it was found to be nonfunctional even in control plants (all vessels were embolized), and because hydraulic and optical VCs were performed in younger stems.

### Xylem sap extraction

In order to compare xylem sap properties in L and S plants under drought and in the following recovery phase, xylem sap was collected from L_C_, S_C_, L_Dr_, S_Dr_, L_Rec_ and S_Rec_ plants using a vacuum extraction technique (Secchi & Zwieniecki, 2012). Because L_Dr_ saplings were less vulnerable than S_Dr_ saplings (see the Results section), five additional L plants were harvested at the Ψ_xyl_ of S_Dr_ plants (*c*. −1.25 MPa; from here on, L_–1.25 MPa_) to measure PLC and xylem sap properties. Briefly, a 2 ml vial was inserted into a small vacuum chamber, connected to a vacuum pump and sealed with a rubber stopper with an inserted syringe needle. The proximal end of the stem was cut with a razor blade, and debarked for a length of 3–4 cm. The cut surface was washed with deionized water using a high‐pressure dental flosser (Apiker, Shenzhen, China) to remove cell debris (Schenk *et al*., 2017). The proximal end was inserted into a high‐density polyethylene tube connected to the needle, and a vacuum of 0.08 MPa was applied. Segments 1 cm in length were progressively cut from the distal end to allow sap flow from the cut open vessels into the tube. After extraction, vials were sealed and immediately stored at −20°C until analyses.

### NSC analyses of wood, bark and xylem sap

Stem samples were separated in wood and bark fractions, microwaved for 3 min at 700 W and oven‐dried at 70°C for 24 h, then grinded with a mixer mill (MM400; Retsch GmbH, Haan, Germany) and stored at −20°C until extraction.

Nonstructural carbohydrate extraction and analysis followed Tomasella *et al*., (2019a). Briefly, 15 mg of powder were suspended in 300 μl of 80% (v/v) ethanol, and incubated in a water bath at 80°C for 30 min. After centrifugation at 11 000 ***g***, the supernatant was collected and the extraction procedure was repeated a second time with the remaining pellet. The extracted solution was evaporated in the oven at 55°C, while the pellet was suspended in 500 μl of 50 mM Tris‐HCl (pH 8.0) and incubated at 80°C in a water bath for 30 min. The supernatant was centrifuged and transferred in the vial with the evaporated sugars, which were carefully resuspended. The remaining pellet was suspended in 1 ml 0.4 M sodium acetate trihydrate (pH 4.6) buffer solution, boiled at 100°C for 1 h and cooled to room temperature. 100 U α‐amylase (from *Aspergillus orizae*; Sigma‐Aldrich) and 25 U amyloglucosidase (from *Aspergillus nigrae*; Sigma‐Aldrich) were added to the solution, which was incubated overnight at 55°C, boiled for 3 min to stop enzymatic activity and centrifuged at 8000 ***g*** for 5 min. Samples were stored at −20°C until analysis.

Soluble NSC (for wood, bark and xylem sap) and starch (for wood and bark) were quantified using the Anthrone method (Yemm & Willis, 1954). Anthrone was dissolved in sulphuric acid in a 1 g l^−1^ solution and kept in the dark. 5–25 μl of sample (10 μl in case of xylem sap) was placed in microplates and the Anthrone reagent was added to reach 200 μl solution and mixed. The microplates were placed on ice for 10 min, then in the oven at 100°C for 20 min, and finally cooled to room temperature for 20 min. Absorbance was read at 620 nm in a Multilabel Plate Reader (Victor 3; PerkinElmer Inc., Waltham, MA, USA). Standard solutions of glucose and amylose were used for calibration curves converting absorbance values to glucose/amylose concentrations for soluble NSC and starch content estimation, respectively. Amylose underwent the same extraction procedure described for the pellet of samples for starch analysis.

### Xylem sap surface tension and pH

Frozen sap samples were transported to the University of Innsbruck and stored at −20°C until analysis. After thawing at room temperature, xylem sap samples were put in an ultrasonic bath (Elmasonic S100; Elma Schmidbauer GmbH, Singen, Germany) for 3 min to remove eventual particle aggregates that formed during freeze–thaw (not present when sap was extracted). About 20–40 μl of sap were sampled with a syringe, and sap surface tension (*γ*_sap_, mN m^−1^) was measured with a video‐based optical contact angle measuring system (OCA 15EC; DataPhysics Instruments GmbH, Filderstadt, Germany), using the pendant‐drop method. Depending on available sap volumes, one to three drops per sample were measured and the average *γ*_sap_ was calculated. Immediately after *γ*_sap_ measurements, xylem sap pH was measured with a pH meter (Twin pH Meter B‐212; Horiba, Kyoto, Japan).

### Xylem anatomy

Anatomical analyses were performed in air‐dried stem samples used for hydraulic measurements of L_Dr_ and S_Dr_ plants, to check for structural determinants of recorded differences in hydraulic vulnerability among groups (see the Results section).

Seven stems per treatment were softened in a 99% ethanol : glycerol : distilled water solution (1 : 1 : 1) for 4 d. Transverse sections 20 μm thick were then prepared with a sledge microtome (Sledge Microtome G.S.L.1; Schenkung Dapples, Zuerich, Switzerland), stained with a mixture of safranin and alcian blue solution (35 : 65) and observed under a light microscope. Pictures were taken with a camera (Progress Gryphax; Jenoptik AG, Jena, Germany) at ×20 magnification (Olympus BX41) on a radial sector of sapwood, including all annual rings. Analyses were conducted on a stem sector, including all rings and, separately, only in the last year ring (i.e. the ring formed in the year when shading was applied). Vessel mean arithmetic (*D*) and hydraulic (*D*_h_) diameter, vessel density (VD), ‘thickness to wall span ratio’ ((*t*/*b*)_h_^2^), vessel grouping index (*V*_G_), and the vesselled area (i.e. the percentage of sapwood occupied by vessels) were measured following Scholz *et al*. (2013).

For SEM measurements, five air‐dried stem samples per treatment were softened for 4 d in distilled water. For each sample, a transverse and a radial section (both about 3 mm thick) were obtained with a sledge microtome (see earlier) and by hand with a razor blade, respectively. Samples were initially dehydrated following an ethanol series (10–30–50–75%) for 30 min in each solution, then left for 3 h in a 75% EtOH‐formaldehyde dimethyl acetal (FDA) solution (1 : 1) and for 12 h in FDA. Complete dehydration was reached by using a critical point dryer (CPD 030; Bal‐Tec, Balzers, Liechtenstein). Samples were mounted onto stubs with double‐sided tape and electron‐conductive carbon cement (LEIT‐C; Plano GmbH, Wetzlar, Germany), dried for 24 h in the oven at 40°C and finally sputter‐coated with gold (EMSCD 050; Leica Microsystems, Wetzlar, Germany). Samples were observed with a scanning electron microscope (EVO 10; Carl Zeiss Microscopy GmbH, Jena, Germany) under 15 kV accelerating voltage. Pictures were taken at 1000–2000× magnification, in pit fields located as close as possible to the cambium, but at a maximum distance of 200 μm (average thickness of the last year ring). In the radial sections, a minimum of 40 pits included in a minimum of five pit fields per sample were analysed. Maximum and minimum pit apertures (*D*_PA_max_, *D*_PA_min_) and pit chamber diameters (*D*_PC_max_, *D*_PC_min_) were measured. Pit aperture area (*A*_PA_) and chamber area (*A*_PC_) were obtained from minimum and maximum diameters through the formula of ovals, and pit aperture fraction (*F*_PA_ = *A*_PA_/*A*_PC_) was calculated (Lens *et al*., 2011). From tangential sections, the pit chamber depth (Depth_PC_) was measured. A minimum of five pits (10 pits on average) per sample were analysed. All anatomical measurements were performed with imagej.

### Biomass, stem diameter and height

Plant height (*h*) and stem basal diameter (*d*) were measured at plant transplant, the day before applying the shading treatment and at harvest at the end of the experiment (August).

Relative growth rate in terms of height (RGR_h_) and stem diameter (RGR_d_) were calculated as:

$$\text{RGR}=\left(h\;\text{or}\;d\;\text{at harvest}‐h\;\text{or}\;d\;\text{before shading}\right)\times {\left(h\;\text{or}\;d\;\text{before shading}\right)}^{‐1}.$$

In addition, roots, leaves and stems were harvested and oven‐dried at 70°C for 24 h and weighed to obtain root, leaf, stem and shoot (leaf + stem) dry biomass, namely *B*_root_, *B*_leaf_, *B*_stem_ and *B*_shoot_.

### Statistical analyses

Hydraulic and optical VCs were fitted in sigmaplot to a four‐parameter sigmoidal function:

$$f=y0+a/\left(1+\exp\left(‐\left(x‐x0\right)/b\right)\right)$$

where *y*0 is the minimum fitted PLC value, *a* is a constrained parameter so that *a* + *y*0 = 100 (i.e. 100% PLC or embolized pixels), *x*0 is the inflection point (steepest point of the curve, corresponding to Ψ_50_ if *y*0 were 0), *b* is a parameter related to the slope of the curve. This function was used because plants were found to have residual embolized conduits in the older year rings (see also micro‐CT pictures), and therefore PLC of well‐hydrated plants was > 0%. For *x*0, differences between L and S curves for each method as well as between methods were determined based on 95% confidence interval (CI) overlap.

All other statistical analyses were carried out with R (R Core Team, 2017). Boxplot panels were obtained with the ggplot2 package. For all analysed parameters, when assumptions of normality of residuals and homogeneity of variances were not violated, one‐way ANOVA tests (response variable ~ *f*(treatment)) through the ‘aov’ function were run, followed by Tukey's honestly significant difference (HSD) *post hoc* test (only for significant ANOVA, *α* = 0.05) through the ‘TukeyHSD’ function in the stats package. When homogeneity of variances assumption was violated, generalized least‐squares (GLS) models were calculated with the ‘gls’ function including the ‘varIdent’ variance structure, in the R package nlme (Pinheiro *et al*., 2016), and followed by Tukey's HSD *post hoc* analysis (for significant tests), with p‐values adjusted using the Bonferroni‐Holm method.

## Results

On all monitoring dates (with exception of 30 July) *g*_L_ was significantly lower in S_C_ than in L_C_ plants, averaging 137 ± 13 and 377 ± 51 mmol m^−2^ s^−1^, respectively (Fig. 2b). Ψ_xyl_ was similar in the two groups and ranged between −0.7 and −1.1 MPa (Fig. 2c).

### Xylem vulnerability to embolism

Hydraulic measurements suggested the presence of native embolism in well‐hydrated plants, as PLC was *c*. 26% in both L and S plants (Fig. 3a). Native embolism could not be captured by the optical method, as this technique detects only embolism events occurring during measurements. This methodological difference accounts for the difference in native embolism levels between hydraulic and optical VCs (Fig. 3a,b). Both hydraulic and optical methods showed that S plants were more vulnerable to xylem embolism than L plants (Fig. 3a,b), with a significant shift in the inflection point (*x*0) of 0.30 and 0.22 MPa for the hydraulic and optical methods, respectively. In the hydraulic VCs, average *x*0 values were −1.45 and − 1.15 MPa, and in the optical VCs they were −1.36 and − 1.14 MPa in L and S plants, respectively. Given that the minimum value of the function was not PLC = 0, the inflection point *x*0 did not correspond to Ψ_xyl_ at 50% PLC (Ψ_50_), but to Ψ_xyl_ at 65% and 55% PLC for the hydraulic and optical VCs, respectively. Overall, hydraulic and optical VCs were similar except for *x*0 of L plants that was slightly (0.09 MPa) but significantly more negative in the hydraulic curve than in the optical curve (Fig. 3a,b; Table S1).

The shift in xylem vulnerability induced by shading was confirmed by X‐ray micro‐CT, as the percentage of embolized xylem area (*A*_embol_) at the target Ψ_xyl_ of −1.4 MPa was higher in S than in L plants (Figs 3d,e, S2), that is, 5.1% and 7.3% vs 3.3% and 3.8% (single individual values), respectively. These *A*_embol_ values are in agreement with those detected in poplar by Secchi *et al*. (2021). Micro‐CT scans also confirmed, for well‐watered plants (Ψ_xyl_ = −0.3 MPa on average), the presence of residual embolized conduits as inferred from hydraulic VCs (Fig. 3c).

### Xylem anatomy

Anatomical analyses of L_dr_ and S_dr_ stems did not reveal differences in any of the measured parameters, considering either all the year rings together (Table S2) or only the current‐year ring (Table 1). Average *D* was 26.5 μm and average (*t*/*b*)*_h_* ^2^ was 8.4 × 10^−3^. SEM analyses of pit characteristics in the current‐year ring did not reveal any significant shade‐induced modification. However, despite similar *A*_PA_ and *A*_PC_, their ratio (*F*_PA_) tended to be slightly higher in S_Dr_ than in L_Dr_ plants (0.12 and 0.10, respectively; *P* = 0.07).

**Table 1:** Wood anatomical parameters measured in the last year ring of drought‐stressed light (L_Dr_) and shaded (S_Dr_) *Populus nigra* plants.

	L_Dr_	S_Dr_	*P‐*value
*D* (µm)	26.3 ± 0.9	26.9 ± 0.9	0.622
*D*_h_ (µm)	37.6 ± 1.4	37.3 ± 1.3	0.871
(*t/b*)_h_^2^ × 10^−3^	8.27 ± 0.78	8.65 ± 1.13	0.798
VD (mm^−2^)	401 ± 46	374 ± 27	0.619
*V*_G_	1.60 ± 0.07	1.62 ± 0.10	0.848
Vesselled area (%)	24.8 ± 2.9	23.7 ± 0.7	0.612
*D*_PA_max_ (µm)	2.82 ± 0.15	3.03 ± 0.19	0.396
*D*_PA_min_ (µm)	1.72 ± 0.04	1.95 ± 0.11	0.091
*A*_PA_ (µm^2^)	3.9 ± 0.3	4.8 ± 0.6	0.180
*D*_PC_max_ (µm)	7.70 ± 0.07	7.61 ± 0.21	0.704
*D*_PC_min_ (µm)	6.80 ± 0.08	6.65 ± 0.12	0.301
*A*_PC_ (µm^2^)	41.3 ± 0.7	40.0 ± 1.7	0.497
*F*_PA_	0.10 ± 0.01	0.12 ± 0.01	0.070
Depth_PC_ (µm)	1.29 ± 0.09	1.19 ± 0.05	0.371

*D*, vessel mean arithmetic diameter; *D*_h_, vessel mean hydraulic diameter; (*t/b*)_h_^2^, ‘thickness to wall span ratio’; VD, vessel density; *V*_G_, vessel grouping index; vesselled area, percentage of sapwood occupied by vessels; *D*_PA_max_, maximum pit aperture diameter; *D*_PA_min_, minimum pit aperture diameter; *A*_PA_, pit aperture area; *D*_PC_max_, maximum pit chamber diameter; *D*_PC_min_, minimum pit chamber diameter; *A*_PC_, pit chamber area; *F*_PA_, pit aperture fraction; Depth_PC_, pit chamber depth. Values are means ± SE.

### Xylem hydraulics and water potential

**Fig. 4:**
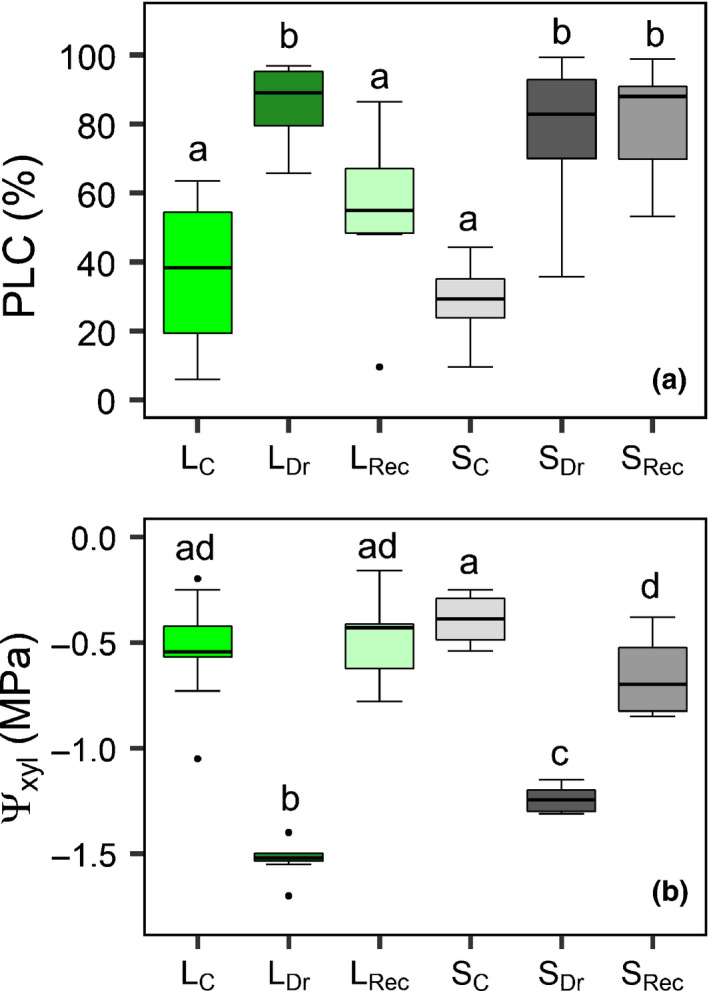
Percentage loss of hydraulic conductance (PLC, a) and xylem water potential (Ψ_xyl_, b) measured in light (L) and shaded (S) control (L_C_, S_C_), drought (L_Dr_, S_Dr_) and recovery (L_Rec_, S_Rec_) *Populus nigra* plants. Boxplots provide the median (horizontal line) and interquartile ranges. Single points are the outliers. Different letters indicate significant differences among groups (*P* < 0.05).

Native PLC was similar in L_C_ and S_C_ plants. According to their different vulnerabilities, L_Dr_ and S_Dr_ plants reached the target PLC (*c*. 80%), that is, 85.9 ± 4.3% and 77.7 ± 7.5%, respectively, at different Ψ_xyl_, that is, at −1.53 ± 0.03 and − 1.24 ± 0.02 MPa, respectively (Fig. 4a,b).

After 3 d of re‐irrigation, PLC recovered in L_Rec_ plants (PLC = 54%), but not in S_Rec_ plants. Ψ_xyl_ significantly increased in both treatments, but returned to control values only in L_Rec_ plants, whereas in S_Rec_ plants it was, on average, still 0.3 MPa lower than the respective controls.

### Stem NSC content

**Fig. 5:**
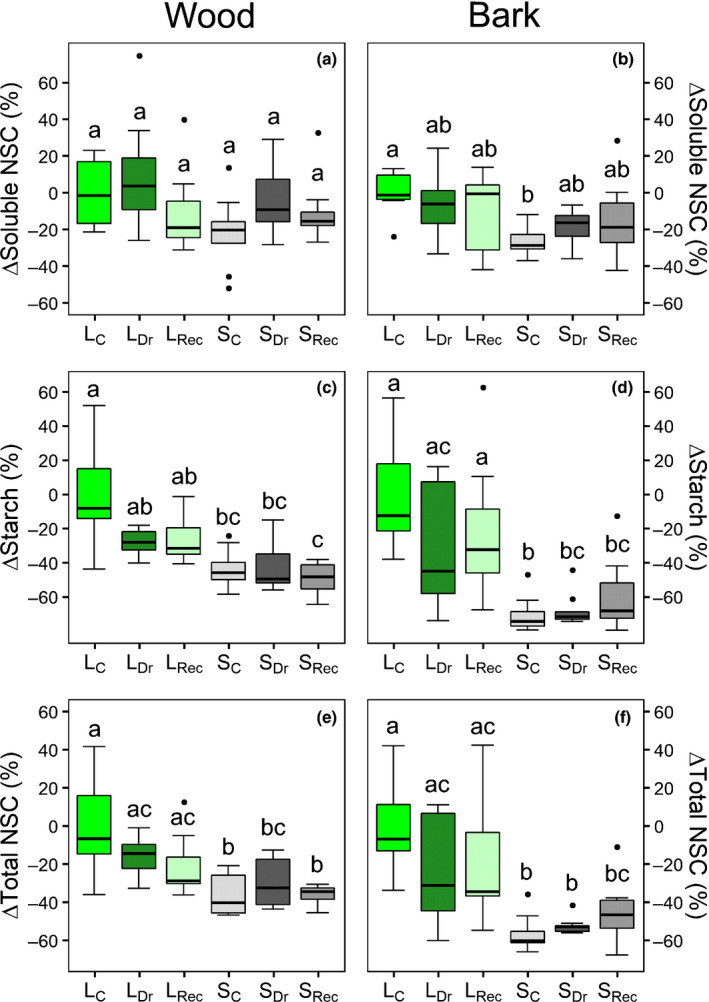
Percentage stem nonstructural carbohydrate (NSC) variation (Δ) with respect to control light (L_C_) plants. ΔSoluble NSC (a, b), ΔStarch (c, d) and ΔTotal NSC (e, f) measured in wood (a, c, e) and bark (b, d, f) of light (L) and shaded (S) control (L_C_, S_C_), drought (L_Dr_, S_Dr_) and recovery (L_Rec_, S_Rec_) *Populus nigra* plants. Boxplots provide the median (horizontal line) and interquartile ranges. Single points are the outliers. Different letters indicate significant differences among groups (*P* < 0.05).

The shading treatment significantly depleted NSCs in both wood and bark, except for soluble NSC content in the wood that remained almost constant in all treatments and hydration stages (Figs 5, S3). In particular, starch and total NSC concentrations were always significantly lower in shaded control (S_C_) than in light control (L_C_) plants, in both bark and wood. Shading lowered total NSC by about 30% and 50% in wood and bark, respectively. In S plants starch concentration in the bark decreased by 50–70% with respect to L plants in all groups, approaching values similar to those measured in the wood (Fig. 5c,d). Differences between the two drought‐stressed groups were not significant, except for total bark NSC, again lower in S_Dr_ than in L_Dr_ plants.

### Xylem sap sugar content, pH and surface tension

**Fig. 6:**
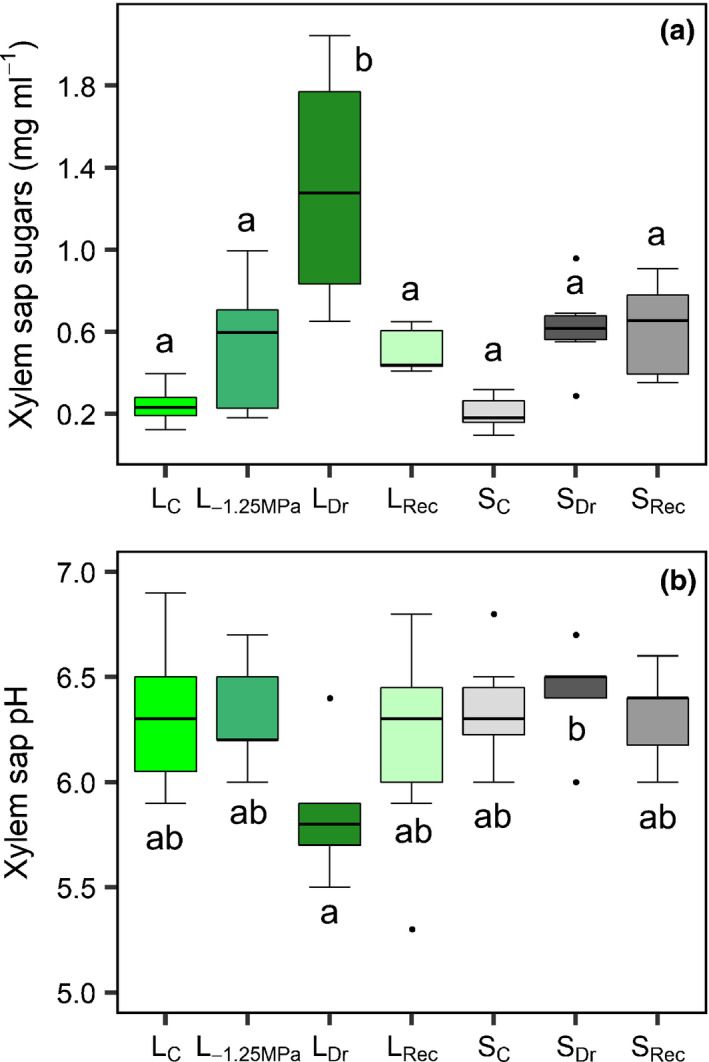
Xylem sap sugar concentration (a) and pH (b) measured in light (L) and shaded (S) control (L_C_, S_C_), drought (L_Dr_, S_Dr_) and recovery (L_Rec_, S_Rec_) *Populus nigra* plants. L_–1.25 MPa_ indicates L plants stressed to −1.25 MPa (i.e. at the same water potential of S_Dr_ plants). Boxplots provide the median (horizontal line) and interquartile ranges. Single points are the outliers. Different letters indicate significant differences among groups (*P* < 0.05).

Xylem sap sugar concentration was *c*. 0.2 mg ml^−1^ in both L_C_ and S_C_ plants and increased (not significantly) to *c*. 0.6 mg ml^−1^ in all drought and recovery groups except for L_Dr_ plants, where the increase was much higher and significant, reaching 1.2 mg ml^−1^ (Fig. 6a).

Xylem sap pH was similar (6.3 on average) between all treatments except for L_Dr_ plants, in which it dropped to 5.9 and was significantly different with respect to S_Dr_ plants (*P* = 0.02; Fig. 6b).

*γ*_sap_ ranged between 70 and 73 mN m^−1^ and did not differ between treatments (Fig. S4).

### Biomass, stem diameter and height

The shading treatment strongly impacted biomass production and relative growth rate (*P* < 0.001; Table S3). *B*_stem_ and *B*_root_ were one‐fold, *B*_shoot_ was two‐fold and *B*_leaf_ three‐fold higher in L_C_ than in S_C_ plants. L_C_ plants, in about 60–70 d, from the beginning of the shading period to the harvest at the end of the experiment, had grown by *c*. 32% and 29% in height and basal diameter, whereas S_C_ plants grew by only 6% and 5%, respectively.

## Discussion

Our data provide new insights into the impact of NSC availability on xylem hydraulics, sap chemistry, and post‐drought hydraulic recovery. Shade‐induced reduction of photosynthesis strongly impacted stem NSC (mainly starch) content which was, in turn, accompanied by: increased xylem hydraulic vulnerability; lack of xylem sap sugar accumulation and acidification under drought; and lack of hydraulic recovery upon re‐irrigation. Our results highlight the importance of stem NSC in hydraulic functioning under fluctuations in water supply.

### Prolonged shading reduces stem NSC concentration and increases xylem vulnerability

Hydraulic VCs were in agreement with optical VCs (Fig. 3). In L plants, the slight (0.09 MPa) difference in the inflection point of the curve (*x*0) between the methods (Table S1) depends on the fact that the optical method could be applied only to current‐year stems, while hydraulic measurements were performed on 1‐yr‐old segments. Moreover, the optical method does not quantify PLC, as done instead by hydraulic methods (Venturas *et al*., 2019). The overall consistency between these methods is in agreement with recent reports (Gauthey *et al*., 2020), and raises confidence regarding the lack of artefacts deriving from sample preparation for hydraulic measurements (Wheeler *et al*., 2013; Trifilò *et al*., 2014; Venturas *et al*., 2015). Most importantly, the two methods converged in revealing higher vulnerability in S than in L plants, and X‐ray micro‐CT scans supported these results. This shift in vulnerability is in agreement with previous observations based on acoustic emissions in shaded stems of poplar (De Baerdemaeker *et al*., 2017).

Shading impacted plant growth (as shown by RGR_h_ and RGR_d_), as well as root and shoot biomass production. Therefore, we initially hypothesized that xylem anatomy of S plants could also be substantially different from that of L plants, thus affecting the vulnerability to embolism. However, anatomical features of vessels and ultrastructure of pits were not different between L and S saplings (Table 1). *F*_PA_ tended to be slightly higher in S_Dr_ than in L_Dr_ plants. Accordingly, F_PA_ positively correlated with xylem vulnerability in *Acer* species (Lens *et al*., 2011), suggesting that smaller F_PA_ might provide more mechanical support to aspirated pit membranes (Klepsch *et al*., 2018): future experiments could test this hypothesis. Shading can affect xylem function through effects on thickness of intervessel pit membranes, as thinner pit membranes would be a weaker barrier against air seeding (Jansen *et al*., 2009). This was shown in conifers grown in the understorey compared with full sunlight (Schoonmaker *et al*., 2010), and in 3‐month‐old saplings grown in the shade (Plavcová *et al*., 2011). We could not measure pit membrane thickness because stems were air‐dried before analysis, a procedure known to produce artefacts when measuring this trait (Plavcová *et al*., 2011). However, the most likely cause for production of less resistant xylem in shaded trees would be the exposure to higher Ψ_xyl_ under lower irradiance (Lemoine *et al*., 2002). In our experiment Ψ_xyl_ was similar in the two treatment groups along the shading period, albeit it tended to be slightly lower in L_C_ than in S_C_ plants (Fig. 2c). However, the Ψ_xyl_ of all well‐watered plants was overall moderate and above vulnerability thresholds, possibly explaining the lack of anatomical adjustments. Moreover, we think it unlikely that the small xylem area built by S plants during the experimental period could have produced a large impact on its vulnerability.

Besides anatomy, sap characteristics can affect xylem vulnerability to embolism (Tyree & Zimmermann, 2002). In timberline conifers, *γ*_sap_ underwent seasonal fluctuations with related impacts on hydraulic safety (Losso *et al*., 2017). Similarly, in fungal‐infected Norway spruce, lower xylem hydraulic conductivity was accompanied by decreased *γ*_sap_, and by increased concentration of several organic compounds in the sap (Paljakka *et al*., 2020). In our plants, *γ*_sap_ was very similar to pure water, as observed in other studies (Christensen‐Dalsgaard *et al*., 2011), and it did not change across treatments (Fig. S4). Lipids and proteins in the xylem sap have recently been suggested to act as surfactants and stabilizers of gas nanobubbles (Schenk *et al*., 2017), and De Baerdemaeker *et al*. (2017) hypothesized that shade‐induced inhibition of leaf/stem photosynthesis might reduce the synthesis of these compounds. We cannot exclude this hypothesis, and further studies on the influence of NSC on the synthesis and presence of lipids and other compounds in the xylem sap are needed.

In our study, shading was applied to the whole plant, thus limiting photosynthesis at both leaf and stem levels. Therefore, we cannot disentangle the relative importance of leaf vs stem photosynthesis in the maintenance of stem hydraulics. Recent studies have shown that stem photosynthesis is crucial for local supply of NSCs during drought (De Roo *et al*., 2020). In our saplings, NSC concentration was already strongly decreased after 50 d of shading in well‐watered plants, and not only under drought conditions. This could be a result of the fact that PPFD in S plants was low enough (close to the light compensation point; Zhao *et al*., 2015) to minimize growth and NSC storage. Moreover, the small size of saplings and low total NSC storage pools could had been exhausted faster than in larger trees (Weber *et al*., 2018). This strong NSC depletion, similarly to *Acer* seedlings exposed to complete darkness (Piper & Fajardo, 2016), caused strong growth limitations and extremely low biomass production compared with light‐exposed plants.

Besides effects on xylem growth and sap chemistry, NSC depletion could be indirectly involved in the increase in xylem hydraulic vulnerability. Sugar upload in living cells maintains cell turgor and‐continuity between wood parenchyma cells and both phloem cells and xylem conduits. Vessel‐associated cells (VACs), in particular, directly exchange water and other molecules/ions with the xylem apoplast (Morris *et al*., 2018). Under drought conditions, the water potential of these cells probably equilibrates with xylem water potential, possibly leading to cell turgor loss and shrinkage when tension becomes exceedingly high (Oparka, 1994). We suggest that in wood parenchyma of S plants, turgor loss and cell shrinkage might occur at less negative Ψ_xyl_ as a result of lower sugar concentration and osmoregulation capacity. Indeed, in well‐watered *Pinus ponderosa* seedlings shade‐induced NSC depletion impaired osmoregulation and turgor maintenance (Sapes *et al*., 2021). A shift to higher turgor loss point could lead to formation of air gaps between xylem vessels and VACs. We hypothesize that these air pockets would impede the exchange of water and solutes and possibly become additional air seeding sources, increasing the chances for xylem embolism build‐up.

Our study specifically focused on possible NSC effects on stem hydraulics, but it is still unclear whether NSC depletion would similarly influence leaf and root hydraulics. Given that a shift to higher turgor loss point has been observed in NSC‐depleted plants (Sapes *et al*., 2021), it is also possible that the extra‐xylary hydraulic pathways, which can largely contribute to the loss of hydraulic conductance in these organs under drought (e.g. Scoffoni *et al*., 2017), might be impacted by NSC depletion.

### Shading impedes sugar accumulation and acidification of xylem sap at the peak of drought

We observed significant changes of xylem sap chemistry associated with increasing PLC in L_Dr_ plants, suggesting that parenchyma cells were responsible for a drop in apoplastic pH and increased sugar concentration (Secchi & Zwieniecki, 2012; Pagliarani *et al*., 2019; Secchi *et al*., 2021). Accordingly, we found a significant negative linear relationship between xylem sap pH and sugar concentration in L_Dr_ plants (Fig. S5). The pH drop from 6.3 in L_C_ plants to 5.8 in L_Dr_ plants was similar to that observed in drought‐exposed hybrid poplars (Pagliarani *et al*., 2019). Xylem sap acidification has been reported in several other species under drought (Sharp & Davies, 2009; Losso *et al*., 2018) and is supposed to enhance acidic invertase activity and sucrose hydrolysis, therefore decreasing the osmotic potential of xylem sap (Secchi & Zwieniecki, 2016). Considering that these events have been reported to accompany increased PLC, it is remarkable that in our study such changes were detected only in L_Dr_ and not in S_Dr_ plants, despite similar PLC values. We propose two possible, not mutually exclusive, explanations: the cascade of events culminating in sucrose efflux into the xylem sap is not triggered by embolism build‐up, but by an unknown factor, possibly related to light availability; or the low amount of local starch reserves impedes the cascade of metabolic responses, ending with sugar accumulation in xylem sap, and possibly linked to xylem refilling when xylem tension is relieved (Pagliarani *et al*., 2019; Secchi *et al*., 2021). In relation to the latter hypothesis, S plants could have prioritized the delivery of sugars derived from the low leftover starch reserves to target sinks different from xylem sap, such as xylem parenchyma and phloem, to preserve their functionality.

### Shade‐induced NSC depletion impedes hydraulic recovery upon re‐irrigation

In our experiment, only L plants stressed to *c*. 80% PLC (L_Dr_) recovered hydraulic function, whereas S plants experiencing the same PLC (S_Dr_) did not reduce embolism levels after re‐irrigation. The recovery from embolism in L plants under moderate tension is consistent with results obtained by previous studies (Secchi & Zwieniecki, 2012; Pagliarani *et al*., 2019). Our results are also in accordance with findings obtained on *F. ornus* saplings subjected to shade and prolonged drought that, in contrast to plants maintained at ambient light and subjected to fast drought, did not recover their hydraulic function upon re‐irrigation (Tomasella *et al*., 2019a). We exclude the possibility that new functional xylem had been already produced 3 d after re‐irrigation, and specifically only in L_Rec_ plants. In fact, cross‐sections of L_Rec_ and S_Rec_ stems did not show any evidence of new cambial growth (Fig. S6).

According to the xylem refilling model, hydraulic recovery is primed by the pH‐driven accumulation of sugars in the sap (Secchi & Zwieniecki, 2016). Indeed, this cascade of events can be disrupted or delayed by artificially blocking stem metabolic activity or altering xylem sap pH (Salleo *et al*., 2004; Secchi *et al*., 2021). We additionally show that shade‐induced depletion of NSC produces similar effects. In particular, only plants with high stem starch content (Fig. 5), higher sap sugar concentration and lower pH at the end of drought (Fig. 6) did recover hydraulic function upon rehydration (Fig. 4). Embolism reversal detected *in vivo* by micro‐CT scans in a poplar hybrid occurred from the cambium (i.e. in proximity to the phloem) to the inner xylem (Secchi *et al*., 2021). This, together with the observation that our shading treatment impacted NSC content mostly in the bark, could suggest the involvement of NSC stored in the phloem in the recovery process (Nardini *et al*., 2011b).

Liu *et al*. (2019) showed that xylem hydraulic restoration via bark water uptake in water‐soaked stem segments occurred only under light conditions, suggesting that corticular photosynthesis provides the sugars required for osmotically driven water uptake and xylem refilling. An interesting question is whether light plays a role in the hydraulic recovery only as an energy source to allow sugar production, or if it also triggers other important mechanisms. Light is known to enhance leaf aquaporin transcript abundances and activity (Nardini *et al*., 2005; Ben Baaziz *et al*., 2012), and further studies are needed to understand if light favours the stem hydraulic recovery mechanism via effects on aquaporin abundance.

### Conclusions

We have provided novel insights into the role of NSCs in preserving hydraulic function in poplar saplings during and after drought. Increased xylem vulnerability in shaded plants could not be explained by changes in wood anatomical properties, suggesting that stem NSCs are important for maintaining plant hydraulics under drought conditions. In the drought‐induced mortality framework, our data suggest that eventual NSC depletion might not only correlate with hydraulic failure but might even cause it. This could perhaps explain why, in a good number of mortality cases presented by Adams *et al*. (2017), hydraulic failure was accompanied by carbon depletion.

We confirm that pH‐driven sugar accumulation in xylem sap under drought favours embolism recovery after re‐irrigation, but this is impeded by shade‐induced depletion of stem NSCs. We cannot exclude that S trees might have needed a longer time to recover PLC, and it is still unclear if imposing a less intense, longer drought could differentially affect xylem hydraulics and the other stem and xylem sap physiological responses.

Clarifying the role of light in the cell metabolic activity and in processes occurring at the symplast–apoplast interface during drought and after drought relief is a primary objective of future mechanistic studies on xylem embolism recovery.

## Author contributions

MT and AN designed the experiment; MT performed the experiment and measurements, with help from SN and FP; MT, SN and VC performed NSC extraction and analysis; WK and MT performed SEM sample preparation and observation; SM and BB contributed to *γ*_sap_ and anatomy data analyses and discussion. MT and AN analysed the data and wrote the manuscript, with contributions and revisions from all authors.

## Supporting information


**Fig. S1** Daily mean air temperature and relative humidity.
**Fig. S2** Transverse images of stems obtained with micro‐CT.
**Fig. S3** Stem NSC concentrations.
**Fig. S4** Xylem sap surface tension (*γ*_sap_).
**Fig. S5** Relationships between xylem sap sugar concentration and pH.
**Fig. S6** Transverse anatomical sections of stems obtained with a light microscope in re‐irrigated light and shaded plants.
**Table S1** Output of hydraulic and optical vulnerability curve fitting.
**Table S2** Wood anatomical parameters measured in drought‐stressed light (L_Dr_) and shaded (S_Dr_) plants over the whole transverse section (all tree rings).
**Table S3** Plant biomass, relative height and relative diameter growth rate.
Please note: Wiley Blackwell are not responsible for the content or functionality of any Supporting Information supplied by the authors. Any queries (other than missing material) should be directed to the *New Phytologist* Central Office.
