# Supplementary material for: Shade‐induced reduction of stem nonstructural carbohydrates increases xylem vulnerability to embolism and impedes hydraulic recovery in Populus nigra
Source: New Phytol. 2021 May 15;231(1):108–21. doi: 10.1111/nph.17384 (PMC9290559; doi:10.1111/nph.17384)
Supplement: Supplementary file 1 — Fig. S1 Daily mean air temperature and relative humidity. Fig. S2 Transverse images of stems obtained with micro‐CT. Fig. S3 Stem NSC concentrations. Fig. S4 Xylem sap surface tension (γ sap). Fig. S5 Relationships between xylem sap sugar concentration and pH. Fig. S6 Transverse anatomical sections of stems obtained with a light microscope in re‐irrigated light and shaded plants. Table S1 Output of hydraulic and optical vulnerability curve fitting. Table S2 Wood anatomical parameters measured in drought‐stressed light (LDr) and shaded (SDr) plants over the whole transverse section (all tree rings). Table S3 Plant biomass, relative height and relative diameter growth rate. Please note: Wiley Blackwell are not responsible for the content or functionality of any Supporting Information supplied by the authors. Any queries (other than missing material) should be directed to the New Phytologist Central Office. [file NPH-231-108-s001.pdf]

New Phytologist **Supporting Information**

Article title: **Shade-induced reduction of stem non-structural carbohydrates increases xylem vulnerability to embolism and impedes hydraulic recovery in *Populus nigra* L.**

Authors: Martina Tomasella, Valentino Casolo, Sara Natale, Francesco Petruzzellis, Werner Kofler, Barbara Beikircher, Stefan Mayr, Andrea Nardini

Article acceptance date: 28 March 2021

The following Supporting Information is available for this article:

Fig. S1 Daily mean air temperature and relative humidity

Fig. S2 Transverse images of stems obtained with MicroCT

Fig. S3 Stem NSC concentrations

Fig. S4 Xylem sap surface tension (γ_{sap})

Fig. S5 Relationships between xylem sap sugar concentration and pH

Fig. S6 Transverse anatomical sections of stems obtained with a light microscope in re-irrigated plants

Table S1 Output of hydraulic and optical vulnerability curves fitting

Table S2 Wood anatomical parameters measured in drought stressed light (L_{Dr}) and shaded (S_{Dr}) plants over the whole transverse section (all tree rings)

Table S3 Plant biomass, relative height and diameter growth rate

Fig. S1 Daily mean air temperature (a) and relative humidity (RH, b) measured over time from plant transplant to the end of the experiment outside (L treatment) and inside (S treatment) the shading net. The two dataloggers were kept always in the same position in the greenhouse but, when the shading treatment began (indicated by the vertical dashed line), the S datalogger was included under the shading net.

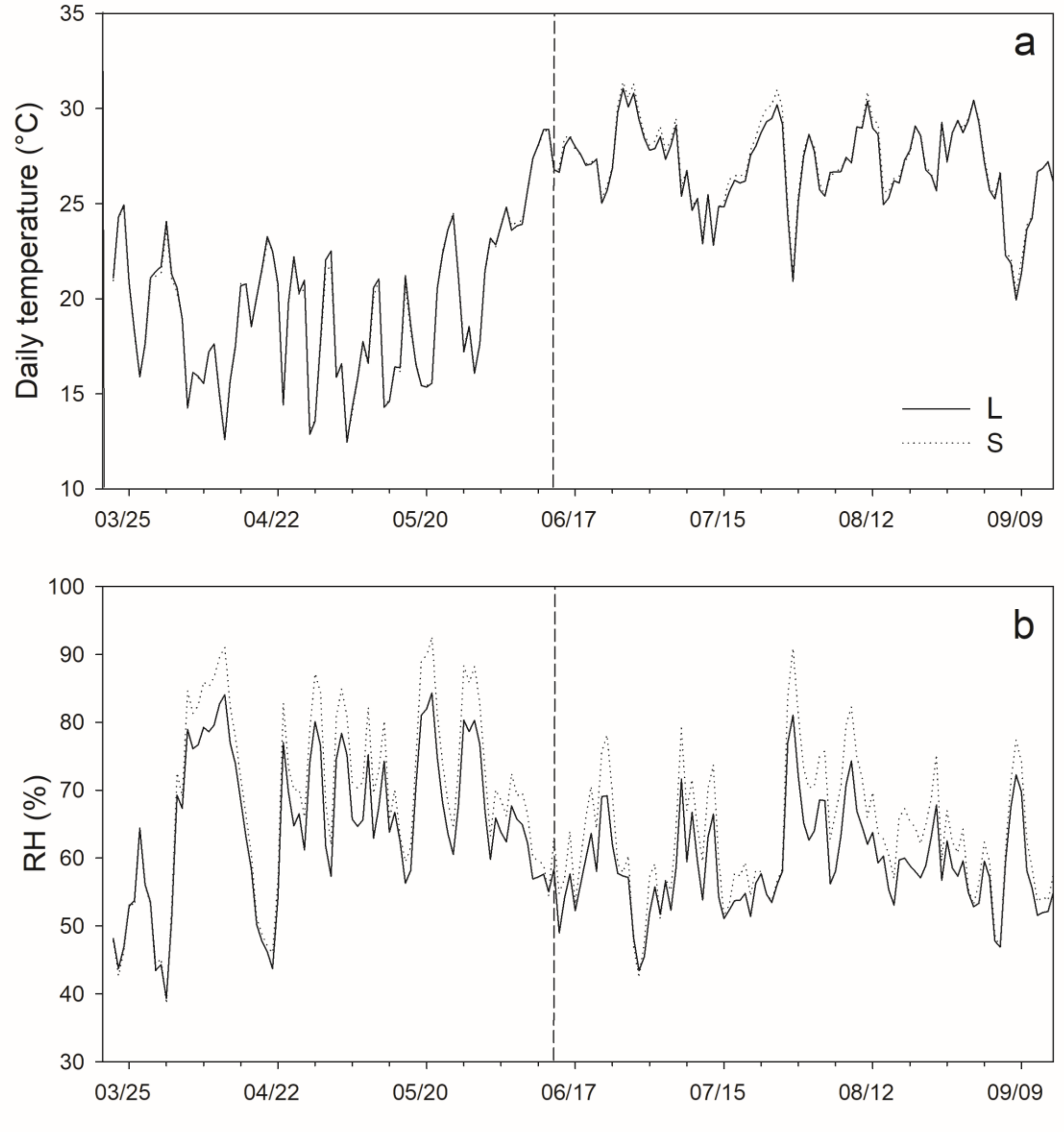

Fig. S2 Transverse images of stems obtained with MicroCT in intact light (L) and shaded (S) plants well irrigated (C) and stressed to ~ -1.40 MPa (Dr). In each image the xylem water potential (Ψ_{xyl}) and the respective percentage of embolized sapwood area (A_{embol}) are reported.

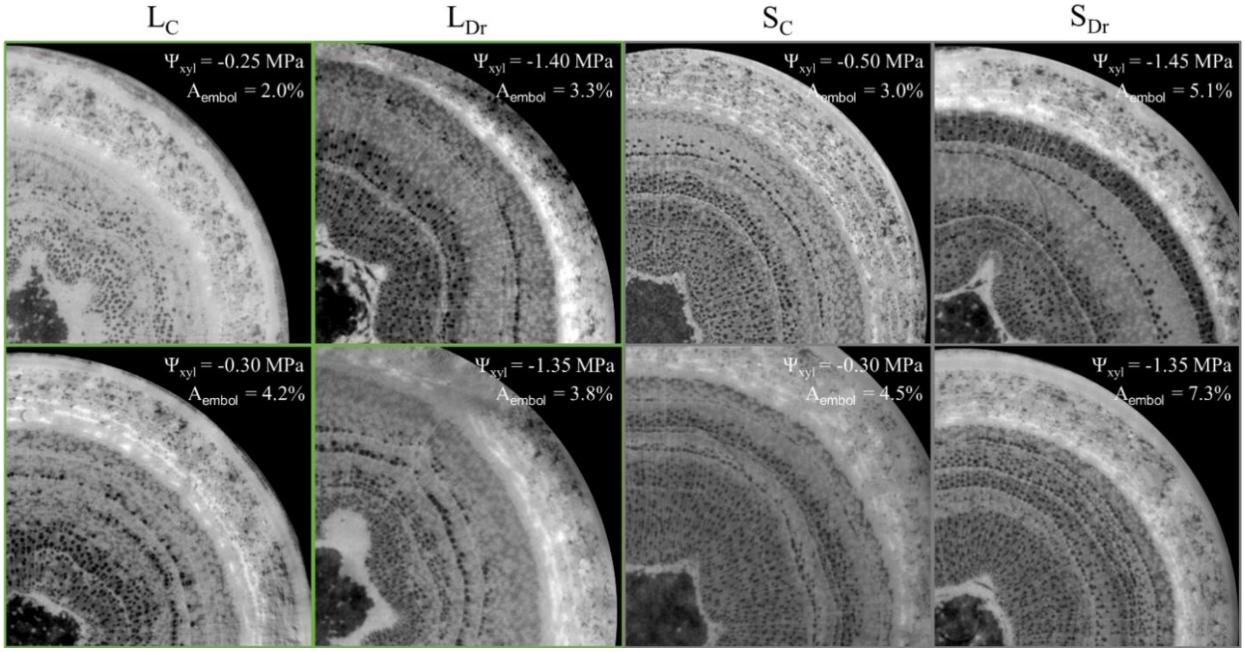

Fig. S3 Stem NSC concentrations. Soluble NSC (a, b), starch (c, d) and total NSC (e, f) measured in wood (a, c, e) and bark (b, d, f) of light (L) and shaded (S) control (L_C, S_C), drought (L_{Dr}, S_{Dr}) and recovery (L_{Rec}, S_{Rec}) plants. Smaller panels in a, c and e report NSC concentrations at a bigger scale. Boxplots provide the median (horizontal line) and interquartile ranges. Single points are the outliers. Different letters indicate significant differences among groups ($P < 0.05$).

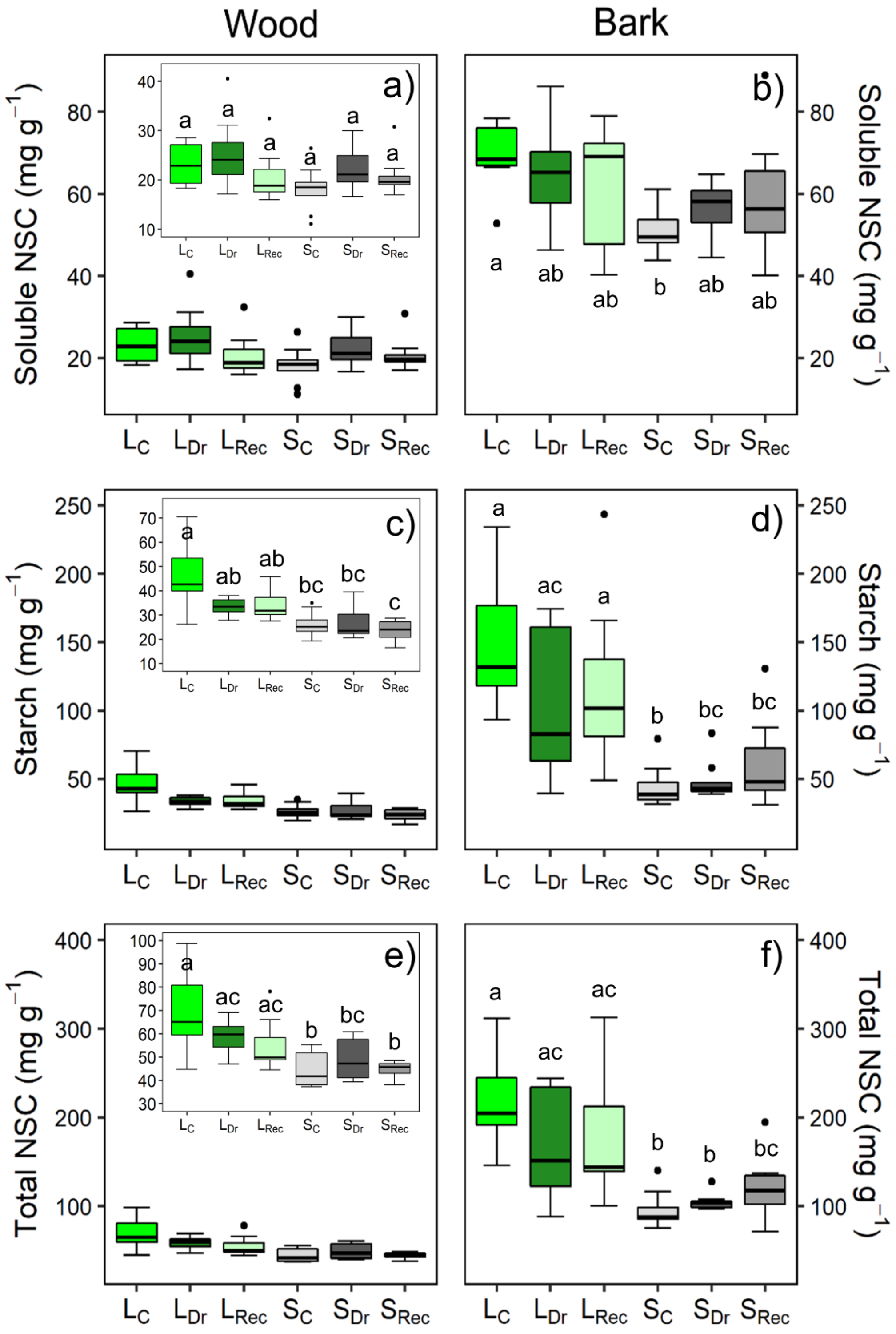

Fig. S4 Xylem sap surface tension (γ_{sap}) measured in light (L) and shaded (S) control (L_C, S_C), drought (L_{Dr}, S_{Dr}) and recovery (L_{Rec}, S_{Rec}) plants. $L_{-1.25MPa}$ indicates L plants stressed to -1.25 MPa, i.e. at the same xylem water potential of S_{Dr} plants. Boxplots provide the median (horizontal line) and interquartile ranges. Single points are the outliers. n.s. = not significant (P > 0.05).

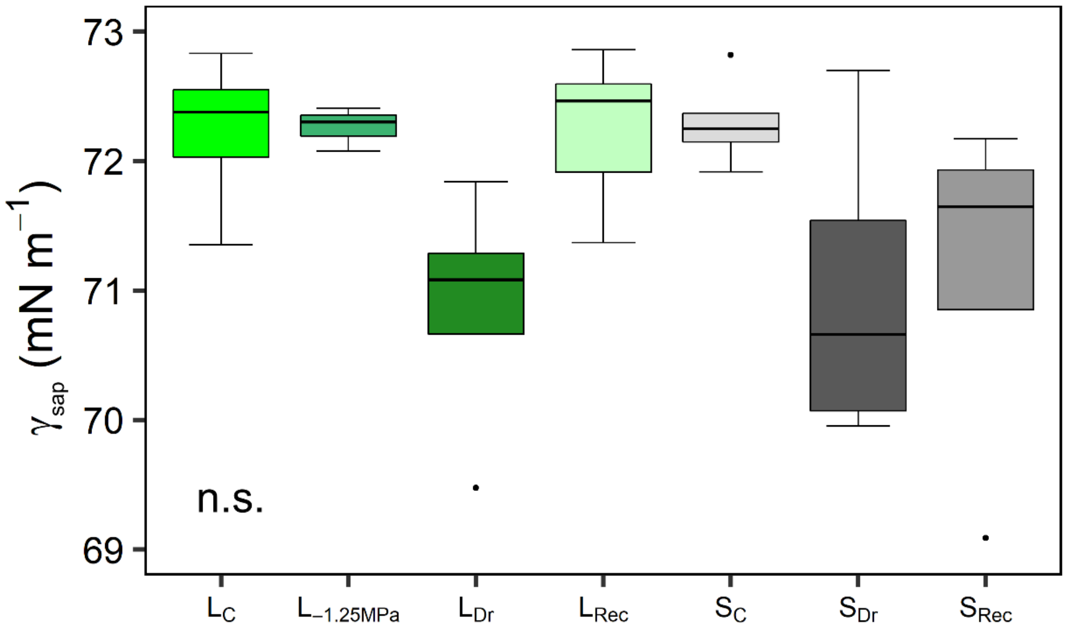

Fig. S5 Relationships between xylem sap sugar concentration and pH in light (L, green) and shaded (S, grey) control (L_C, S_C) and drought (L_{Dr} + $L_{D\text{-}1.25MPa}$, S_{Dr}) plants. The regression line and 0.95 CI resulting from "lm" function (R package 'ggplot2') are reported only for L_{Dr} + $L_{D\text{-}1.25MPa}$ (the only significant, $P < 0.05$) and S_{Dr} ($P = 0.07$) plants. The table on the right shows R^2 and *P*-value of linear regression model ("lm" function, R package 'stats') run for each group.

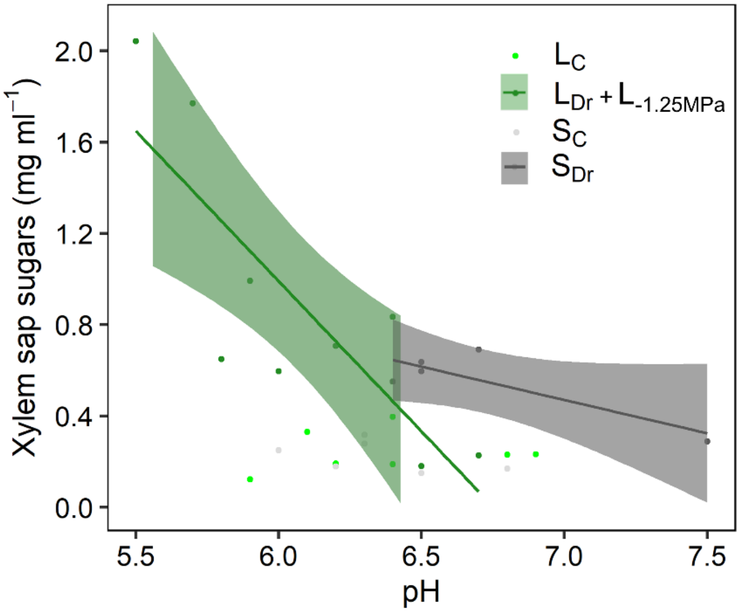

	R^2	*P*
L_C	0.17	0.72
L_{Dr} + $L_{-1.25MPa}$	0.68	0.006
S_C	0.24	0.32
S_{Dr}	0.70	0.07

Fig. S6 Transverse anatomical sections of stems obtained with a light microscope in re-irrigated light and shade plants. Columns show different individuals (n=3). In the wood layers next to the cambium no evident signs of regrowth are visible in light plants with respect to shade plants.

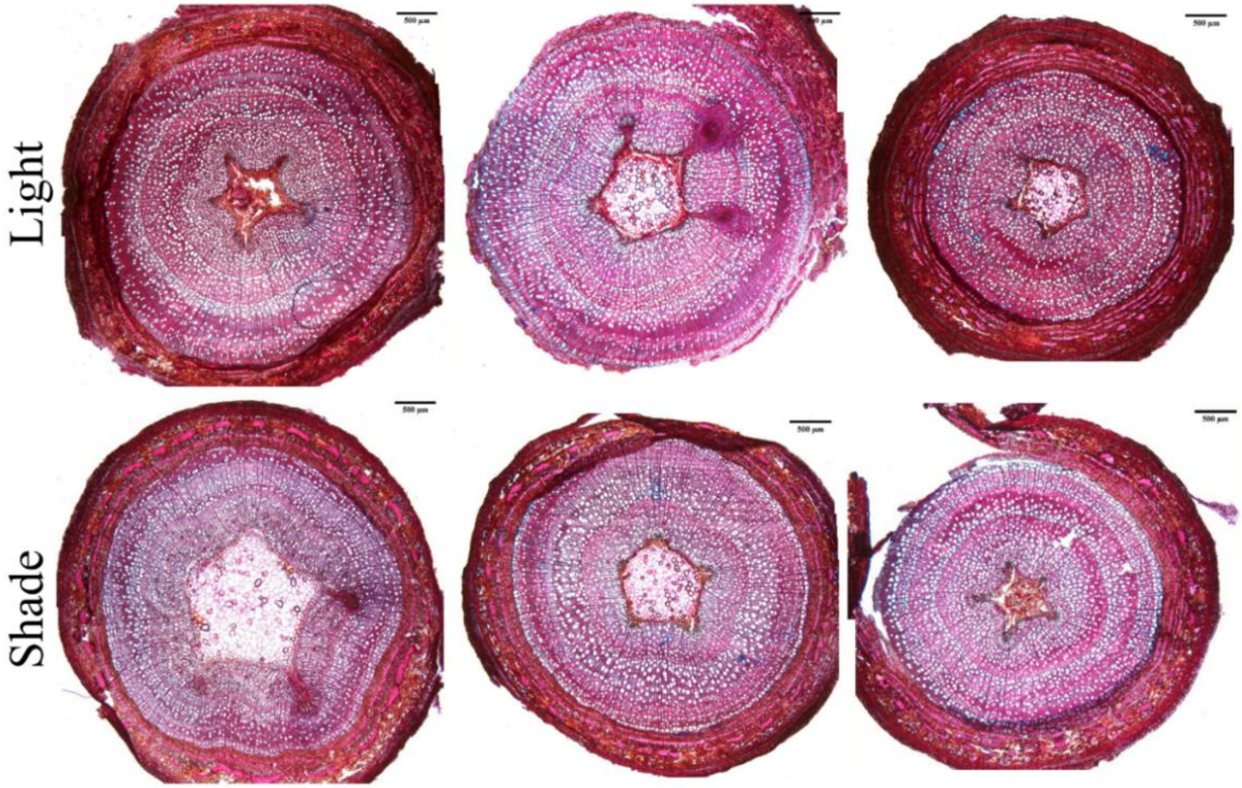

Table S1 Output of hydraulic and optical vulnerability curves fitting for light (L) and shaded (S) plants. Values are means ± SE. The 95% lower and upper bounds of confidence intervals are shown in brackets.

	Hydraulic		Optical	
	L	S	L	S
a	74.5 ± 3.7<br>(66.9, 82.2)	73.2873 ± 4.7<br>(63.8, 82.9)	93.3 ± 2.8<br>(87.6, 99.0)	94.0 ± 3.1<br>(87.7, 100.4)
b	-0.05± 0.02<br>(-0.09,-0.01)	-0.11 ± 0.03<br>(-0.17,-0.04)	-0.06 ± 0.01<br>(-0.08,-0.03)	-0.10 ± 0.01<br>(-0.13,-0.07)
x0	-1.45 ± 0.02<br>(-1.49,-1.41)	-1.15 ± 0.03<br>(-1.22,-1.07)	-1.36 ± 0.01<br>(-1.39,-1.33)	-1.14 ± 0.02<br>(-1.18,-1.10)
y0	25.4± 3.7<br>(17.8, 33.1)	26.7 ± 4.7<br>(17.1, 36.3)	6.7 ± 2.8<br>(1.0, 12.4)	5.9 ± 3.1<br>(-0.4, 12.3)
R^2	0.83	0.77	0.90	0.93

Table S2 Wood anatomical parameters measured in drought stressed light (LDr) and shaded (SDr) plants over the whole transverse section (all tree rings). D = vessel arithmetic diameter; Dh = vessel hydraulic diameter; VD = vessel density; (t/b)h2 = 'thickness to wall span ratio'; VG = vessel grouping index; Vesselled area = percentage of sapwood occupied by vessels. Values are means ± SE.

	L_{Dr}	S_{Dr}	P value
D (µm)	24.9 ± 0.5	25.1 ± 0.4	0.804
D_h (µm)	35.8 ± 0.8	36.7 ± 1.2	0.801
$(tb)_h^2 \times 10^{-3}$	8.12 ± 0.65	8.06 ± 0.34	0.922
VD (mm^{-2})	436 ± 30	409 ± 11	0.425
V_G	1.61 ± 0.03	1.72 ± 0.05	0.089
Vesselled area (%)	24.1 ± 1.9	23.0 ± 0.7	0.611

Table S3 Leaf (Bleaf), stem (Bstem), shoot (Bshoot) and root (Broot) dry biomass, relative height (RGRh) and diameter (RGRD) growth rate measured in light and shaded control trees (LC and SC, respectively) at the end of the experiment. Values are means ± SE. Asterisks indicate significant differences among treatments (P<0.05).

	B_{leaf} (g)	B_{stem} (g)	B_{shoot} (g)	B_{root} (g)	RGR_h	RGR_D
L_C	11.0 ± 0.7*	18.9 ± 2.3*	29.9 ± 2.8*	8.1 ± 0.9*	0.32 ± 0.08*	0.29 ± 0.04*
S_C	2.7 ± 0.4	7.8 ± 0.8	10.5 ± 1.2	3.7 ± 0.3	0.06 ± 0.01	0.05 ± 0.01
